# CD133 significance in glioblastoma development: in silico and in vitro study

**DOI:** 10.1186/s40001-024-01754-2

**Published:** 2024-03-06

**Authors:** Mahdi Abdoli Shadbad, Fatemeh Nejadi Orang, Behzad Baradaran

**Affiliations:** 1 Student Research Committee, Tabriz University of Medical Sciences, Tabriz, Iran; 2 Immunology Research Center, Tabriz University of Medical Sciences, Daneshgah St, Tabriz, Iran

**Keywords:** CD133, Glioblastoma, Temozolomide, PI3K/Akt, MAPK

## Abstract

**Background:**

Glioblastoma multiform (GBM) is among the commonly diagnosed brain malignancies with poor prognosis. CD133 has been introduced as an oncogene in various cancers, like GBM. This study aimed to investigate the significance of *CD133* in GBM development using in silico and in vitro techniques.

**Method:**

The TCGA-GBM database was analyzed for the correlational and comparative studies. After selecting the U87MG cell line, CD133-siRNA was transfected into U87MG cells and treated with temozolomide. The cell viability, cell cycle, migration, clonogenicity, and apoptosis of groups were investigated using MTT, flow cytometry, wound-healing, colony formation, and annexin V/PI assays. Using qRT-PCR method, the mRNA expression levels of *MMP16*, *SOX2*, *RAF1*, *MAP2K1*, *MAPK3*, *PIK3CA*, *AKT3*, *mTOR*, *CDK4*, and *BCL2* were studied.

**Results:**

*CD133* silencing improves apoptosis rate, arrests the cell cycle at the sub-G1 phase, suppresses the clonogenicity of U87MG cells, and inhibits the PI3K/Akt and MAPK pathways via downregulating the *RAF1*, *MAP2K1*, *MAPK3*, *PIK3CA*, *AKT3*, and *mTOR* expression. Besides, combining *CD133* silencing with temozolomide treatment considerably inhibits the migration of U87MG cells compared to temozolomide monotherapy.

**Conclusion:**

*CD133* can regulate the PI3K/Akt and MAPK pathways and modulate the clonogenicity, apoptosis, and cell cycle of GBM. Combining *CD133* silencing with temozolomide treatment considerably increases apoptosis, arrests the cell cycle at the sub-G1, and suppresses migration of U87MG cells compared to temozolomide monotherapy.

**Graphical Abstract:**

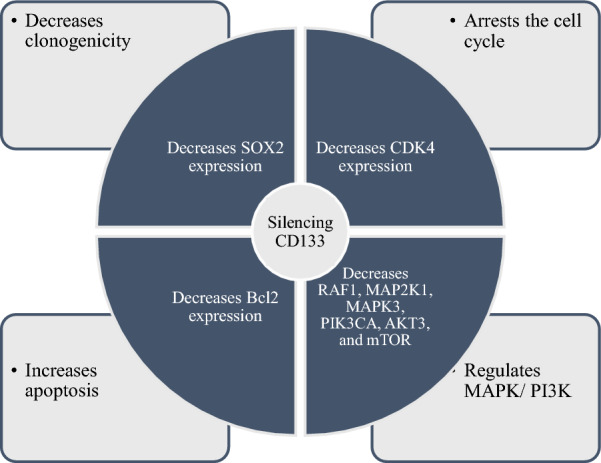

## Introduction

Glioblastoma multiform (GBM) remains a commonly diagnosed primary brain malignancy [1]. Overall, the median survival of affected patients is less than 15 months [2]. Chemoresistance and the highly aggressive nature of GBM severely affect the prognosis of affected patients [3]. A better understanding of GBM biology can provide ample opportunity to develop novel treatments for affected patients.

Cancer stem cells are small populations of malignant cells with stem-like properties; their symmetric and asymmetric divisions and self-renewal result in maintaining tumors [4]. Their quiescence nature and efficacy for extensive DNA repair can shield cancer stem cells from chemoradiotherapy [5]. In this regard, it has been reported that glioma stem cells can be associated with tumor recurrence [5, 6]. Therefore, identifying and targeting these cells can provide ample opportunities to improve the prognoses of affected patients.

CD133 is one of the introduced cancer stem cell markers for various cancers like glioma [7]. It has been implicated in the invasion, migration, tumor sphere formation, and chemoresistance of melanoma cells [8]. In hepatocellular carcinoma, CD133 silencing has been associated with inhibited invasion and vascular endothelial growth factor (VEGF) expression, and increased activity of natural killer cells [9]. In animal models, CD133^+^ pancreatic cancer cells produce metastatic nodules; however, CD133 silencing reverses this pro-tumoral effect. It has been found that the Erk1/2 and SRC signaling pathways regulate the CD133 expression, and CD133 can modulate the epithelial-to-mesenchymal transition in pancreatic cancer cells [10]. It has been reported that hypoxia can upregulate CD133 in glioma cells, and CD133 silencing enhances the chemosensitivity of glioma cells to cisplatin [11]. Aside from this, a recent meta-analysis study has shown that CD133 upregulation is associated with sooner distant tumor recurrence in magnetic resonance imaging patients with glioblastoma [12]. Therefore, CD133 can have a significant role in GBM biology.

Although CD133 has been introduced as a cancer stem marker in GBM and a recent meta-analysis has indicated its clinical significance for GBM patients [12], there is no study investigating the significance of CD133 in various aspects of GBM development and the potential impact of CD133 on the oncogenic signaling pathways of GBM. The present study aimed to investigate the significance of CD133 in GBM development and evaluate the combination of *CD133*-silencing with temozolomide on GBM development. This study leveraged the in silico and in vitro techniques to study the potential CD133-mediated pathways implicated in GBM development.

## Materials and methods

### In silico investigation

The cancer genome atlas (TCGA)-genotype-tissue expression (GTEx) dataset was accessed using the GEPIA 2 (http://gepia2.cancer-pku.cn/) to study the expression levels of CD133 in GBM and non-tumoral brain tissues; normal brain tissues were included from the GTEx dataset for the comparison of CD133 expression level. Using the GEPIA 2, the TCGA-GBM was also accessed to investigate the prognostic significance of CD133 in GBM patients; 25% and 75% quartiles were used as criteria for low and high expression of CD133 in the prognostic studies [13], and the TCGA-GBM (Nature 2008) was leveraged to perform correlational studies [14]. The cancer cell line encyclopedia (CCLE) dataset was used to evaluate CD133 expression in GBM cell lines [15], and GeneMANIA (with max resultant genes and attributes of null) and STRING (with a coefficient of 0.1 and maximum additional interactions of null) were used to study the interactions between the studied genes using Cytscape 3.7.0 [16, 17].

### Cell culture

The U87MG, U373, and A172 cell lines were purchased from the Pasture Institute’s cell bank in Tehran, Iran, and cultured in RPMI-1640 supplemented with 10% fetal bovine serum (GIBCO, Carlsbad, CA) and 1% penicillin/streptomycin. The cells were incubated at 37 °C in a 95% humidified, 5% CO_2_ incubator. All of the cell lines were free from mycoplasma contamination.

### Transfection

The U87MG cell line was selected due to its higher expression of CD133 compared to the A172 and U373 cell lines. After reaching 80% confluency, U87MG cells were suspended in a cold electroporation buffer, and the number of cells was counted. 1 × 10^6^ cells were electroporated with scramble, 20, 40, and 60 pmol of CD133-siRNA (Table 1) using a Gene Pulser Electroporation (Bio-Rad, CA, USA) and a 0.4 cm^3^ Gene Pulser Cuvette (Bio-Rad, CA, USA). The utilized pulse type was the square wave, the pulse length was 25 ms, and the voltage was 100 v.

**Table 1:** *CD133*-siRNA sequences

CD133-siRNA	Sense	Anti-sense
CD133 siRNA (a)	5′-UUGUCAUAAUCAAUUUUGGTT-3′	5′-AACAGUAUUAGUUAAAACCTT-3′
CD133 siRNA (b)	5′-UGAAGUUCUGAGCAAAAUCTT-3′	5′-TTGAUUUUGCUCAGAACUUGA-3′
CD133 siRNA (c)	5′-AGAAAGUCCUAUAAUACUCTT-3′	5′-TTGAGUAUUAUAGGACUUUCU-3′

### qRT-PCR

Total RNA from the cells was extracted using the RiboEX reagent (GeneAll Biotechnology, Seoul, South Korea). After that, a spectrophotometer (Thermo Fisher Scientific, Lenexa, South Korea) was used to examine the purity and concentration of the extracted RNA. The complementary DNA (cDNA) was produced using a thermal cycler system and the AddScript cDNA Synthesis Kit (ADDBIO, South Korea). Then, using Thermo Fisher’s StepOnePlusTM Real-Time PCR System, the mRNA expression of *MMP16*, *SOX2*, *RAF1*, *MAP2K1*, *MAPK3*, *PIK3CA*, *AKT3*, *mTOR*, *CDK4*, *BCL2*, and *CD133* was studied. The housekeeping gene was 18s; the pair primer sequences were blasted before the experiment using the NCBI website (https://www.ncbi.nlm.nih.gov/tools/primer-blast/) (Table 2). For the comparison of the mRNA expression levels in the different studied groups, we used the 2^–∆∆Ct^ to obtain the values; then, log 10 of the obtained values were calculated for further statistical analyses.

**Table 2:** The primer sequences of studied genes

Gene	Forward primer	Reverse primer
MMP16	CCCACACCGCTCTATTCCTCC	CCCTGTTGTTTCTCACTCGCC
SOX2	AAAACAGCCCGGACCGCGTC	CTCGTCGATGAACGGCCGCT
RAF1	AGTCACAGCGAATCAGCCTC	GCCTAATTTTGTTTTTCTCCTGGG
MAP2K1	CAATGGCGGTGTGGTGTTC	GATTGCGGGTTTGATCTCCAG
MAPK3	CGCTTCCGCCATGAGAATGT	GGTCAGTCTCCATCAGGTCC
PIK3CA	GAAGCACCTGAATAGGCAAGTCG	GAGCATCCATGAAATCTGGTCGC
AKT3	AGAACGACCAAAGCCAAACACA	AGTCTGTCTGCTACAGCCTGG
mTOR	AGATGCTTGGAACCGGACCTG	CCAAGATGCCACCTTTCCTCTC
CDK4	CCATCAGCACAGTTCGTGAGGT	TCAGTTCGGGATGTGGCACAGA
BCL2	ACTGGCTCTGTCTGAGTAAG	CCTGATGCTCTGGGTAAC
**CD133**	GCTTTGCAATCTCCCTGTTG	TTGATCCGGGTTCTTACCTG
18s	ACCCGTTGAACCCCATTCGTGA	GCCTCACTAAACCATCCAATCGG

### MTT assay

The cell viability and the half-maximal inhibitory concentration (IC50) of temozolomide on U87MG cells was studied using the 3-(4,5-dimethylthiazol-2-yl)-2,5-diphenyl-2*H*-tetrazolium bromide (MTT) assay. After diluting the initial concentration of temozolomide, i.e., 34.3 mg/ml, its diluted concentration was used for treating tumoral cells. Then, 50 μl of MTT (2 mg/ml) (Sigma–Aldrich, M5655) was added to each well of a 96-well plate after 15 × 10^3^ cells had been seeded into each well. The cells were then incubated for 180 min in the incubator. Following the medium removal, the plate was then incubated for 30 min, and 150 μl of dimethyl sulphoxide (DMSO) (Sigma-Aldrich, D4540) was added to each well. After shaking for 10 min, the optical density (OD) of each well was assessed at 570 nm using an ELISA reader (Sunrise RC, Tecan, Switzerland). The formula for calculating each well’s cell viability percentage was as follows: (each value − mean value of DMSO)/(mean of control value − mean value of DMSO) * 100.

### Annexin V/propidium iodide (PI) assay

U87MG cell apoptosis was studied using the annexin V/PI assay. We treated one group of transfected and one group of non-transfected U87MG cells with temozolomide after seeding 2 × 10^5^ transfected and non-transfected cells in a six-well plate; then, we incubated it for 24 h. The cells were stained with annexin V/PI in accordance with the manufacturer’s instructions after a second 24-h incubation (EXBIO, Vestec, Czech Republic). Then, the apoptosis rate of each group was assessed by flow cytometry (MiltenyBiotecTM FACS Quant 10; MiltenyBiotec, Germany). FlowJo was used to analyze the data.

### Cell cycle assay

The seeding and treatment procedures of the annexin V/PI assay were used in this assay as well. Single cells from each group were obtained, fixed with 80% ethanol, and stored at − 20 °C for 24 h. Using flow cytometry (MiltenyBiotecTM FACS Quant 10; MiltenyBiotec, Germany) after RNase treatment and DAPI labeling (Sigma–Aldrich, D9542), cell distribution in the cell cycle phases was studied. The data were analyzed using the FlowJo software (Tree Star, CA, USA).

### Scratch assay

The scratch test was used to investigate the migration of U87MG cells. A 24-well plate was seeded with 3 × 10^5^ U87MG cells. A yellow sterile pipette tip was then used to scrape the cellular monolayer. Using an inverted microscope (Optika, XDS-3, Italy), the related pictures were taken at 0, 12, 24, and 48 h after incubation.

### Colony formation assay

The stemness of the U87MG cells was investigated using the colony formation assay. Crystal violet was used to stain the cells after they had been incubated for 14 days with 3 × 10^5^ transfected and non-transfected cells in a six-well plate. The pictures were taken using an inverted microscope (Optika, XDS-3, Italy).

### Statistical analyses

The in vitro data were analyzed using GraphPad Prism V 8.0.2 (GraphPad Software, CA, USA). The one-way ANOVA was used to perform statistical analyses between more than two groups. The Shapiro–Wilk test was applied to assess the data normality. *P*-values less than 0.05 were regarded as statistically significant. For each experiment, three independent biological replicates were conducted to perform the statistical analyses.

## Results

### In silico investigation on the significance of *CD133* in GBM

**Fig. 1:**
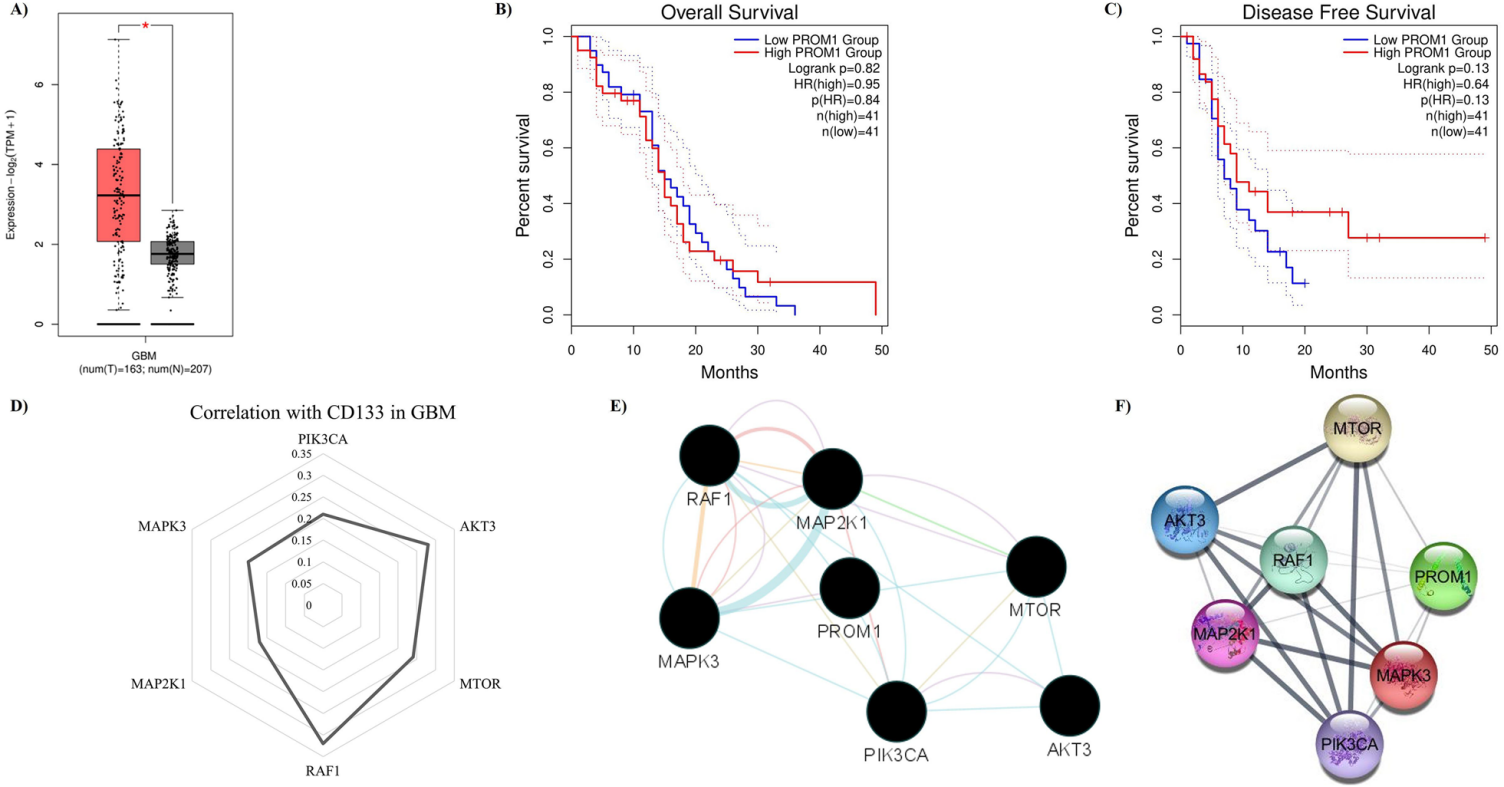
In silico investigation on *CD133* significance in GBM. **A** *CD133* expression in GBM and non-tumoral tissues; *CD133* expression is upregulated in tumoral (T) tissues compared to normal (N) tissues. **B** *CD133* (*PROM1*) expression level is not significantly associated with the overall survival of primary GBM patients. **C** *CD133* (*PROM1*) expression is not significantly associated with the disease-specific survival of primary GBM patients. **D** The correlation between *CD133* and the specific genes of the PI3K/Akt and MAPK pathways. The radar chart represents the correlation coefficient between the studied genes with *CD133* in tumor bulk analysis of GBM tissues. **E** Gene interactions based on GeneMANIA. **F** Gene interactions based on STRING. *PROM1* is the gene symbol name of *CD133*

Firstly, we attempted to study *CD133* expression levels in GBM and non-tumoral brain tissues. Based on the TCGA-GTEX dataset, *CD133* expression is significantly upregulated in GBM tissues (Fig. 1A). The TCGA-GBM was investigated to study the prognostic value of *CD133* in patients with primary GBM as well. The mRNA expression of *CD133* is not significantly associated with the overall survival and disease-specific survival of patients with primary GBM (Fig. 1A, B).

Since CD133 can regulate the PI3k/Akt pathway in prostate adenocarcinoma cells [18], the TCGA-GBM dataset was studied to identify the correlations between *CD133* and the genes of the PI3K/Akt and MAPK pathways. It has been found that there are significant positive correlations between *CD133* with *RAF1*, *mTOR*, and *AKT3* in GBM tissues (Fig. 1C). The GeneMANIA results are in favor of considerable interactions between studied genes (Fig. 1D). These interactions of GeneMANIA are pathways, genetic interactions, predicted, physical interactions, co-expression, and shared protein domains. Consistent with this, the STRING results have also demonstrated considerable interactions between the studied genes (Fig. 1E).

### Selecting a GBM cell line and determining the optimal dose of CD133-siRNA

**Fig. 2:**
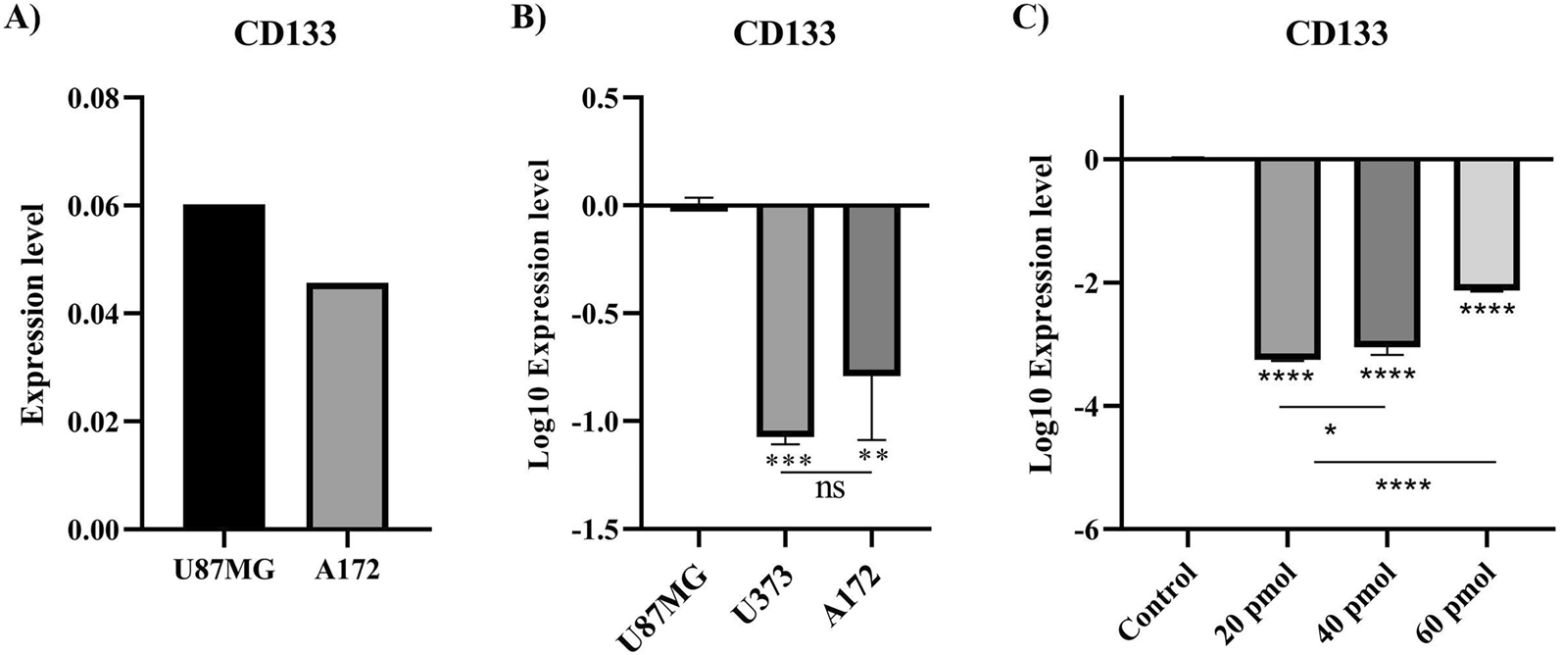
*CD133* expression in GBM cell lines and the optimal dose of *CD133*-siRNA after 48 h of transfection. **A** *CD133* expression level based on the CCLE dataset; U87MG cells have higher *CD133* expression compared to A172 cells. **B** *CD133* expression in A172, U373, and U87MG cells. U87MG cells have higher *CD133* expression compared to A172 and U373 cells. **C** *CD133* expression after 48 h of transfecting CD133-siRNA; 20 pmol CD133-siRNA is the optimal dose for downregulating CD133 in U87MG cells after 48 h. *P-value ≤ 0.05, **P-value ≤ 0.01, ***P-value ≤ 0.001, and ****P-value ≤ 0.0001

Firstly, we leveraged the CCLE dataset to investigate which of the U87MG and A172 cell lines has the highest *CD133* expression. It has been found that there is a trend in *CD133* expression level being higher in the U87MG cell line than the A172 cell line (Fig. 2A). Our experimental results have validated that the *CD133* expression level is significantly higher in U87MG cells compared to A172 and U373 cells (Fig. 2B). Therefore, we used the U87MG cell line for our in vitro experiments. Afterward, we attempted to determine the optimal dose of CD133-siRNA after 48 h of transfection. It has been found that transfection with 20 pmol of CD133-siRNA significantly downregulates the mRNA expression of *CD133* in U87MG cells (*P*-value < 0.0001) (Fig. 2C). However, we were unable to perform flow cytometry studies to investigate the effect of CD133-siRNA on its expression.

### The effect of *CD133* silencing on the cell viability of U87 cells

**Fig. 3:**
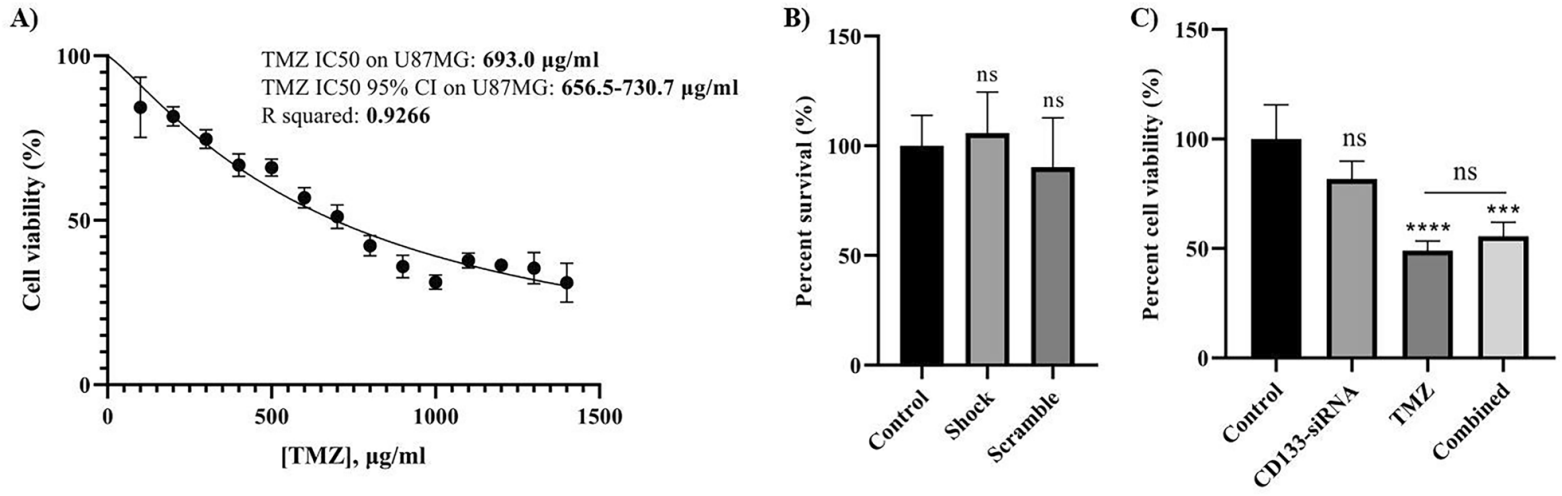
The half-maximal inhibitory concentration (IC50) of temozolomide and the effect of *CD133* silencing on the cell viability of U87MG cells. **A** The IC50 of temozolomide on U87MG cells is 693.0 µg/ml. **B** The effect of shock and scramble transfection on the viability of U87MG cells. The shock of transfection and transfecting scramble is not associated with significant cell viability change. **C** The effect of *CD133* suppression on the cell viability of U87MG cells. *CD133* silencing does not significantly alter the cell viability of U87MG cells. The MTT results are displayed as displayed as mean ± standard deviation (SD). ns: non-significant, ***P-value ≤ 0.001, and ****P-value ≤ 0.0001

We used the MTT assay to determine the effect of CD133-siRNA and the combination therapy on the cell viability of U87MG cells. Firstly, we measured the IC50 of temozolomide on U87MG cells; our results demonstrated that 693 μg/ml is the IC50 of temozolomide on U87MG cells in our experiment (Fig. 3A). To rule out the potential effect of the transfection procedure on the cell viability of U87MG cells, we included the shock and scramble transfection groups as well. Our results have shown that shock and scramble transfection have no statistically significant effect on the cell viability of U87MG cells (*P*-value = 0.7355 and *P*-value = 0.4535, respectively) (Fig. 3B). Also, *CD133* suppression has not significantly altered the cell viability of U87MG cells and has not increased the temozolomide-mediated anti-tumoral effect on the cell viability of U87MG cells (Fig. 3C).

### The effect of *CD133* silencing on the apoptosis of U87MG cells

**Fig. 4:**
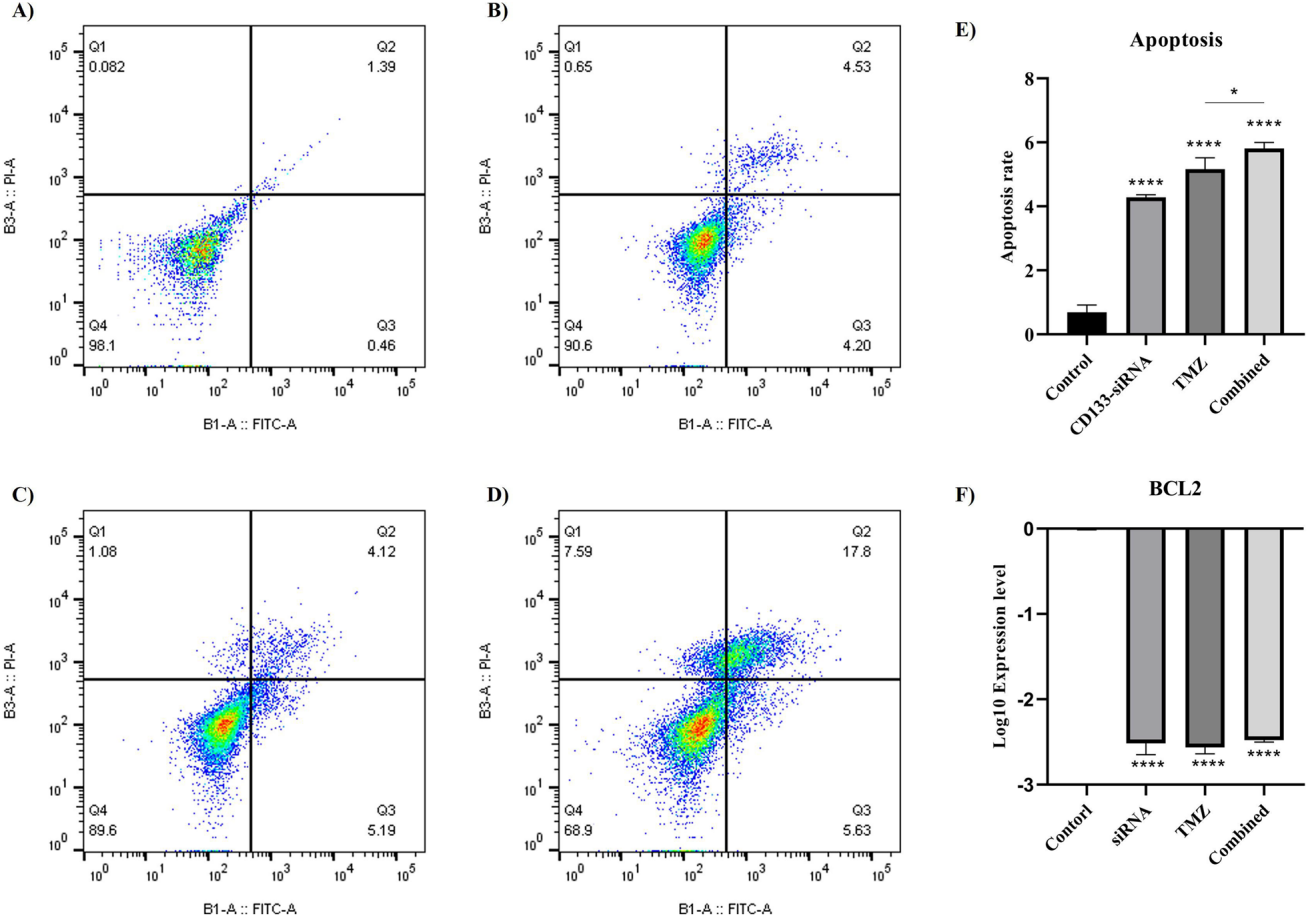
The annexin V/PI assay for evaluating the apoptosis rate of U87MG cells. **A** the annexin V/PI of the control group, **B** the annexin V/PI of the *CD133* silenced group, **C** the annexin V/PI of the temozolomide-treated group, **D** the annexin V/PI of the combined treatment group, **E** the apoptosis rates of studied groups, and **F** the mRNA expression of *BCL2* in different studied groups. *CD133* silencing increases the apoptosis rate of U87MG cells and potentiates the anti-tumoral effect of temozolomide on the apoptosis of U87MG cells. *CD133* silencing downregulates *BCL2* expression in U87MG cells. In the annexin V/PI assay, a lower dose of temozolomide than IC50 was used. *P-value ≤ 0.05, and ****P-value ≤ 0.0001

After evaluating the effect of *CD133* silencing on the cell viability of U87MG cells, we studied its effect on the apoptosis of U87MG cells [19]. Our results have shown that *CD133* silencing significantly improves the apoptosis of U87MG cells (*P*-value < 0.0001). Also, *CD133* silencing potentiates the anti-tumoral effect of temozolomide on the apoptosis of U87MG cells (*P*-value < 0.05). In line with this, *CD133* suppression significantly downregulates *BCL2* expression in U87Mg cells (*P*-value < 0.0001) (Fig. 4). Consistent with this, *CD133* silencing increases the apoptosis of prostate cancer cells and potentiates the paclitaxel-mediated apoptosis in prostate cancer cells [18]. Although *CD133* silencing does not alter the apoptosis of colorectal cancer cells [20, 21], the combined *CD133* silencing with oxaliplatin potentiates the oxaliplatin-mediated apoptosis in colorectal cancer cells [21].

### The effect of *CD133* silencing on the cell cycle of U87MG cells

**Fig. 5:**
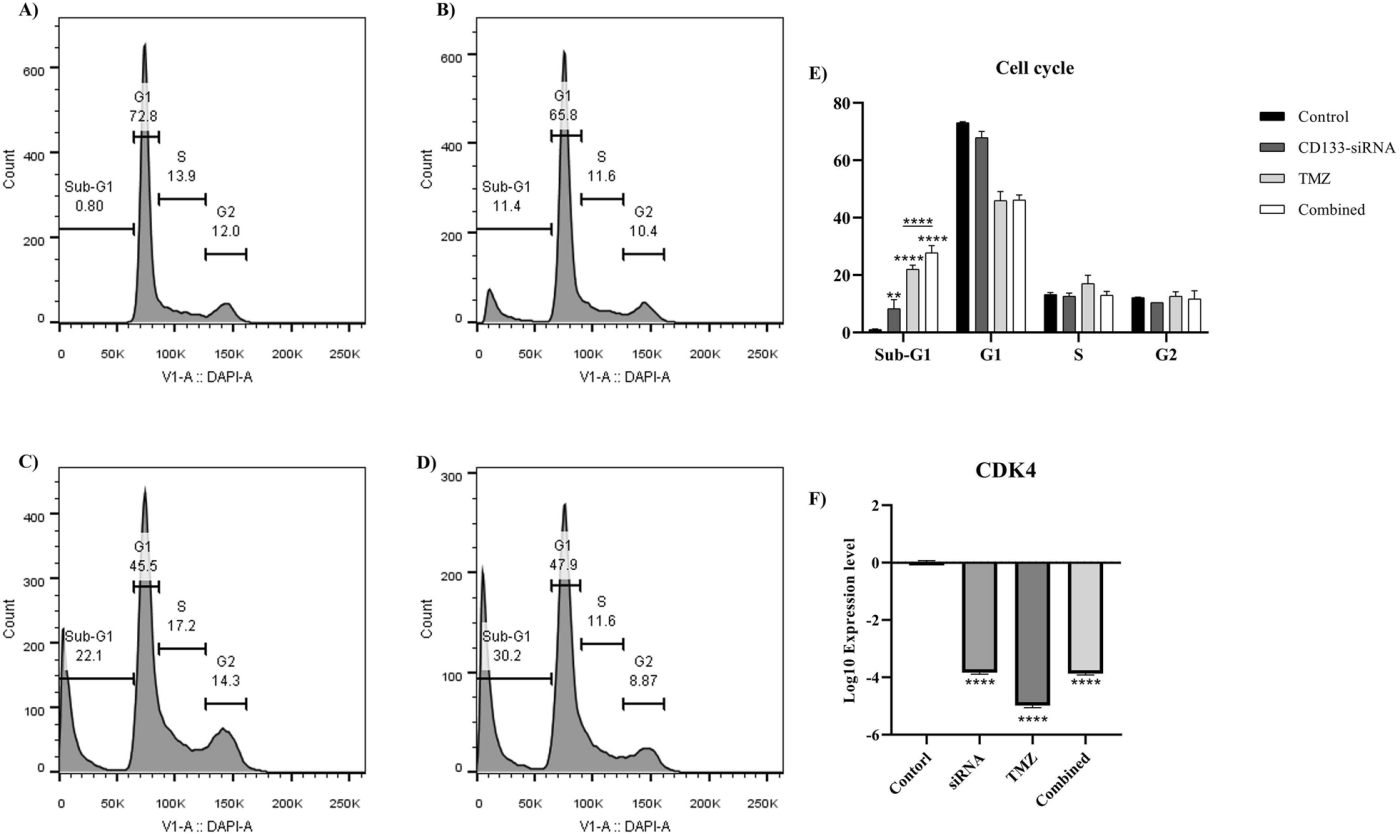
The cell cycle assay for studying the cell cycle phase distribution of U87MG cells. **A** The cell cycle phase distribution of the control group, **B** the cell cycle phase distribution of the *CD133*-silenced group, **C** the cell cycle phase distribution of the temozolomide-treated group, **D** the cell cycle phase distribution of the combined treatment group, **E** the cell cycle phase distribution of studied groups. *CD133* silencing leads to the accumulation of cells at the sub-G1 phase. **F** the mRNA expression of *CDK4* in different studied groups. *CD133* silencing downregulates the expression of *CDK4* in U87MG cells. **P-value ≤ 0.01, and ****P-value ≤ 0.0001

The cell cycle analysis has demonstrated that *CD133* silencing significantly arrests the cell cycle at the sub-G1 phase compared to the control group (*P*-value = 0.0038). Also, the combined therapy of *CD133* silencing with temozolomide significantly arrests the cell cycle compared to monotherapy with temozolomide. In line with these, *CD133* silencing significantly downregulates *CDK4* expression in U87MG cells (*P*-value < 0.0001) (Fig. 5). In prostate cancer cells, the combined *CD133* silencing with paclitaxel arrests the cell cycle at the G2 phase [18]. In colorectal cancer cells, *CD133* silencing arrests the cell cycle of tumoral cells at the G1 phase [21].

### The effect of *CD133* silencing on the migration of U87MG cells

**Fig. 6:**
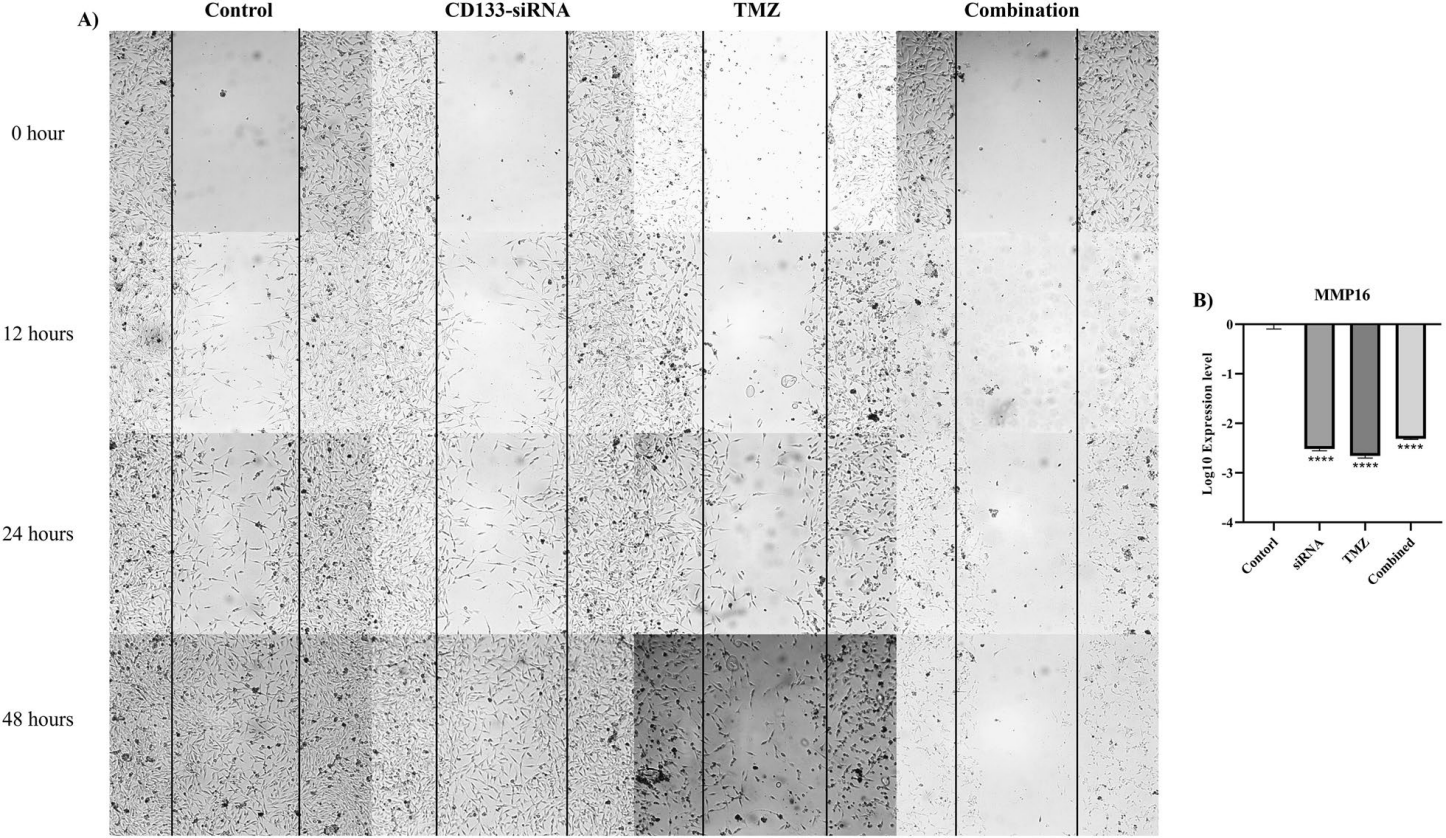
The scratch assay and the mRNA expression of *MMP16*. **A** The scratch assay for assessing the migration of U87MG cells in different studied groups. The combined *CD133* silencing with temozolomide decreases the migration of U87MG cells compared to temozolomide monotherapy. **B** The mRNA expression of *MMP16* in different studied groups. *CD133* silencing downregulates the expression of *MMP16* in U87MG cells. ****P-value ≤ 0.0001

The scratch assay was performed to investigate the effect of *CD133* silencing on the migration of U87MG cells. Our results have shown that the combination therapy decreases the migration of U87MG cells in vitro (Fig. 6A). *CD133* silencing and the combined therapy significantly downregulate *MMP16* expression compared to the control group (*P*-value < 0.0001) (Fig. 6B).

### The effect of *CD133* silencing on the clonogenicity of U87MG cells

**Fig. 7:**
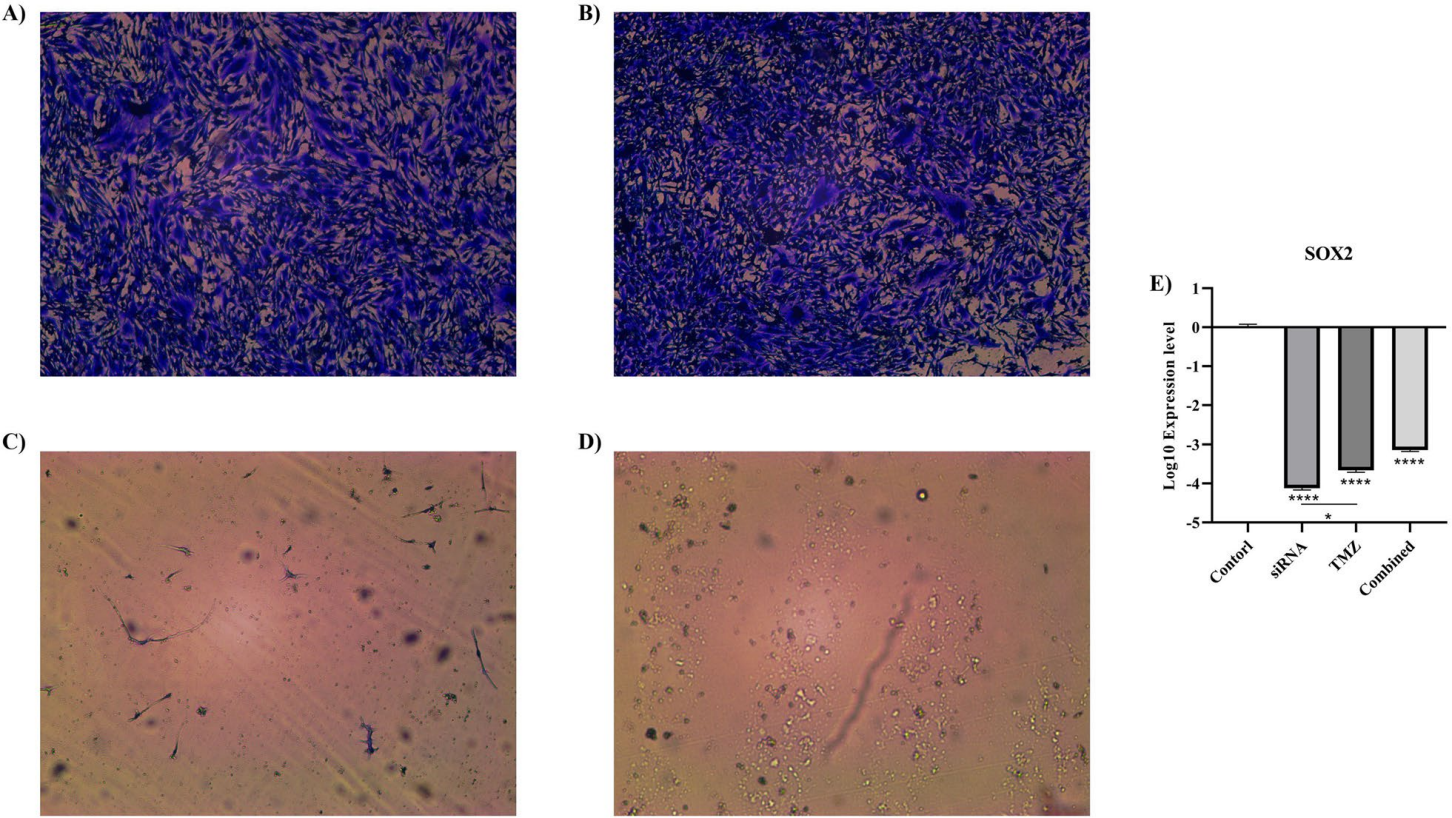
The colony formation and mRNA expression of *SOX2*. **A** The microscopic image of the clonogenicity of the control group. **B** The microscopic image of the clonogenicity of the temozolomide group. **C** The microscopic image of the clonogenicity of the *CD133*-siRNA group. **D** The microscopic image of the clonogenicity of the combined therapy. *CD133* silencing decreases the clonogenicity of U87MG cells. **E** The mRNA expression of *SOX2* in different studied groups. *CD133* silencing downregulates the expression of *SOX2* in U87MG cells. *P-value ≤ 0.05, and ****P-value ≤ 0.0001

The colony formation results have shown that *CD133* silencing decreases the stemness of U87MG cells (Fig. 7A–D). Consistent with this, *CD133* silencing and the combined therapy significantly downregulate *SOX2* expression compared to the control group (both *P*-values ≤ 0.0001) (Fig. 7E).

### *CD133* can regulate the PI3K/Akt and MAPK pathways of U87MG cells

**Fig. 8:**
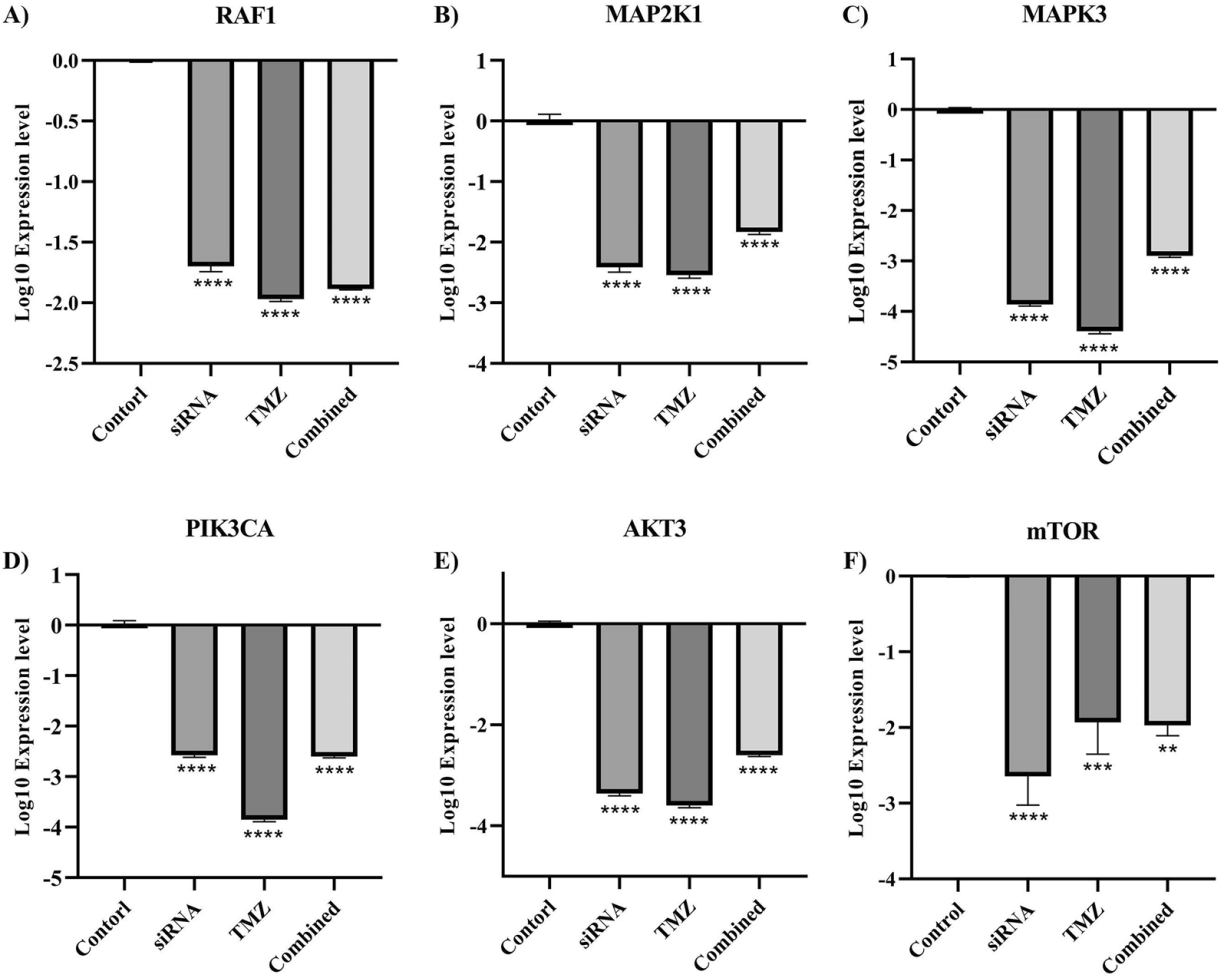
The effect of *CD133* silencing on the signaling factors of the PI3K/Akt and MAPK pathways. **A** The mRNA expression of *RAF1* in different studied groups. *CD133* silencing downregulates *RAF1* expression in U87MG cells. **B** The mRNA expression of *MAP2K1* in different studied groups. *CD133* silencing downregulates *MAP2K1* expression in U87MG cells. **C** The mRNA expression of *MAPK3* in different studied groups. *CD133* silencing downregulates *MAPK3* expression in U87MG cells. **D** The mRNA expression of *PIK3CA* in different studied groups. *CD133* silencing downregulates *PIK3CA* expression in U87MG cells. **E** The mRNA expression of *AKT3* in different studied groups. *CD133* silencing downregulates *AKT3* expression in U87MG cells. **F** The mRNA expression of *mTOR* in different studied groups. *CD133* silencing downregulates *mTOR* expression in U87MG cells. **P-value ≤ 0.01, ***P-value ≤ 0.001, and ****P-value ≤ 0.0001

Following the in silico results, the effect of *CD133* suppression on the studied signaling factors of the PI3K/Akt and MAPK pathways in U87MG was investigated. *CD133* silencing significantly downregulates the expression of *RAF1*, *MAP2K1*, *MAPK3*, *PIK3CA*, *AKT3*, and *mTOR* compared to the control group (Fig. 8). Also, the combination therapy significantly decreases the expression of *RAF1*, *MAP2K1*, *MAPK3*, *PIK3CA*, *AKT3*, and *mTOR* compared to the control group (Fig. 8). However, we could not perform western blot studies to investigate the expression of these factors in protein levels.

## Discussion

The current treatment for primary GBM patients includes tumor resection followed by radiation and temozolomide; tumor-treating field therapy has additional benefits along with temozolomide in treating GBM. Although most cases of primary GBM relapse, there is no well-established therapeutic approach for recurrent GBM [22, 23]. The present study has shown that *CD133* silencing arrests the cell cycle at the sub-G1 phase, improves apoptosis, decreases the clonogenicity of U87MG cells, and can modulate the PI3K/Akt and MAPK pathways via downregulating the *RAF1*, *MAP2K1*, *MAPK3*, *PIK3CA*, *AKT3*, and *mTOR* expression. Also, the combined treatment of *CD133* silencing with temozolomide significantly suppressed the migration of U87MG cells compared to monotherapy with temozolomide.

Initial concepts of brain cancer stem cells were developed in the early 2000s [24–26]. Cancer stem cells are a small population of the tumor bulk that can reproduce original malignant tissue; treatment resistance, tumor recurrence, and low proliferation have been the characteristics of cancer stem cells [4, 27]. It has been shown that cancer stem cells can be responsible for the source of factors associated with poor survival of cancer patients [28]. CD133 has been reported as a cancer stem cell marker in various malignancies [5, 29, 30]. *CD133* is located on chromosome 4p15.33; this glycoprotein is on the cell membrane [31]. Glioma stem cells highly express CD133 compared to differentiated glioma cells [32]. Bresecia et al. have reported that CD133-silencing substantially decreases the clonogenicity in vitro and tumorigenesis in vivo*,* and CD133 re-expression in CD133-silenced cells reverses these anti-tumoral effects in glioma cells [33]. Kang et al. have found that carmustine-resistance GBM cells express CD133, CD117, CD90, CD71, and CD45 and can reconstitute tumor bulk in immunocompromised animal models [34]. Shideng et al. have reported that CD133^+^ glioma cells can effectively activate the repair of radiation-induced DNA damage compared to CD133^−^ glioma cells [35]. Also, the nutrient-deprived tumor microenvironment upregulates CD133 expression, leading to increased survival and decreased apoptosis of glioma cells [36]. CD133 ectopic expression has substantially decreased apoptosis and increased the chemoresistance of glioma cells [37]. Yu et al. have indicated that CD133^+^ glioma stem cells are more invasive than CD133^−^ cells [38]. CD133 protein expression has clinical relevance as well. A recent meta-analysis has shown that increased expression of CD133 is associated with poor progression-free survival in patients with high-grade gliomas and sooner distant tumor recurrence on the MRI of GBM patients [12]. However, our in silico results have shown that there is no statistically significance association between the mRNA expression of CD133 with overall survival and disease-specific survival of primary GBM patients. This might stem from the fact that the mentioned meta-analysis study investigated the protein expression of CD133 rather than the mRNA expression of *CD133*. Despite the scarcity of cancer stem cells in tumor bulk, recent advances in single-cell sequencing approaches have provided ample opportunities to study their genetic properties. Couturier et al. have sorted glioma stem cells into three categories, i.e., CD9^+^/CD133^+^, CD133^−^/CD24^+^/CD9^−^, and CD9^+^/CD44^+^/CD133^−^ glioma cells based on single-cell RNA sequencing. Applying single-cell proteomic analysis and mass cytometry, their results have shown that CD9^+^/CD133^+^ glioma cells implanted in animal models have substantially lower survival than other glioma stem cell subtypes. Indeed, CD9^+^/CD133^+^ glioma cells have higher tumorigenic and temozolomide resistance than other subtypes [39].

The PI3K/Akt and MAPK pathways have been introduced as oncogenic pathways in GBM development [40–42]. Our in silico results have indicated that CD133 can be associated with the signaling factors of PI3K/Akt and MAPK pathways; this hypothesis was strengthened by the evidence of CD133’s impact on the PI3K/Akt pathway in prostate cancer [18]. Our in vitro results have indicated that CD133 can regulate the PI3K/Akt and MAPK pathways, and *CD133* silencing downregulates *RAF1*, *MAP2K1*, *MAPK3*, *PIK3CA*, *AKT3*, and *mTOR* in U87MG cells. In this regard, Dong et al. have reported that CD133 ectopic expression does not affect the proliferation and migration of U87MG cells; however, CD133 ectopic expression increases the phosphorylated Erk and activates the MAPK/Erk pathway in U87MG cells [43]. Wei et al. have reported that CD133^−^ glioma cells are not capable of producing glioma in immunocompromised animal models in contrast to CD133^+^ glioma cells; it has been found that the PI3K activity and the phosphorylations of Akt on S473 and T308 are considerably increased in CD133^+^ population [44]. Furthermore, the results of the performed assays have shown that *CD133* silencing increases apoptosis rate, arrests the cell cycle at the sub-G1 phase, and suppresses the clonogenicity of U87MG cells; however, *CD133* silencing does not significantly affect the cell viability and migration of U87MG cells. Also, our MTT assay has demonstrated that *CD133* silencing does not significantly affect the temozolomide-mediated cytotoxicity on U87MG cells. In line with this, Ahmed et al. have reported that CD133 silencing does not increase the chemosensitivity of U251 cells to temozolomide [11]. Also, it has been reported that *CD133* silencing does not decrease the cell viability of colorectal cancer cells [21]. In prostate cancer, *CD133* silencing does not affect the cell viability of malignant cells [18]. Altogether, CD133 can activate the MAPK/Erk and PI3K/Akt pathways in GBM and regulate the apoptosis, cell cycle, and colonogenicity of GBM cells.

The present study has some limitations. First, we could not perform protein-based assays to investigate the protein expression of the studied genes. Second, we could not include in vivo experiments. Therefore, the obtained results should be interpretated considering these limitations. However, the current study has some strengths as well. First, the hypothesis of our study is developed from in silico investigations. Second, we studied the effect of the combined treatment of temozolomide with *CD133* silencing on various aspects of GBM development, along with the molecular mechanism. Third, we performed a deep literature review to integrate the findings with the obtained results. Collectively, this study provides novel insights into the CD133-mediated molecular mechanisms in GBM development.

In conclusion, this study has shown that CD133 can regulate the PI3K/Akt and MAPK pathways in GBM, and its silencing results in the arrested cell cycle at the sub-G1 phase, stimulated apoptosis, and inhibited clonogenicity of U87MG cells. Although *CD133* silencing does not significantly affect the migration and cell viability of U87MG cells, its combination with temozolomide potentiates the anti-tumoral effects of temozolomide on migration, apoptosis, and cell cycle of U87MG cells.

## Data Availability

The datasets supporting the conclusions of this article are available in the TCGA-GEPIA (http://gepia.cancer-pku.cn/), and cBioPortal (https://www.cbioportal.org/), and CCLE (https://sites.broadinstitute.org/ccle/).
